# The Role of Structural Defects in the Growth of Two-Dimensional Diamond from Graphene

**DOI:** 10.3390/nano12223983

**Published:** 2022-11-12

**Authors:** Liubov A. Varlamova, Sergey V. Erohin, Pavel B. Sorokin

**Affiliations:** Laboratory of Digital Material Science, National University of Science and Technology MISIS, 4 Leninskiy Prospekt, 119049 Moscow, Russia

**Keywords:** bilayer graphene, diamane, hydrogenation, chemically induced phase transition, defects, grain boundary, DFTB

## Abstract

The presented work is devoted to the study of the formation of the thinnest diamond film (diamane). We investigate the initial stages of diamond nucleation in imperfect bilayer graphene exposed by the deposition of H atoms (chemically induced phase transition). We show that defects serve as nucleation centers, their hydrogenation is energy favorable and depends on the defect type. Hydrogenation of vacancies facilitates the binding of graphene layers, but the impact wanes already at the second coordination sphere. Defects influence of 5|7 is lower but promotes diamondization. The grain boundary role is similar but can lead to the final formation of a diamond film consisting of chemically connected grains with different surfaces. Interestingly, even hexagonal and cubic two-dimensional diamonds can coexist together in the same film, which suggests the possibility of obtaining a new two-dimensional polycrystal unexplored before.

## 1. Introduction

Diamond is probably the best-known crystalline compound of carbon. Diamond nanostructures of different dimensions have also attracted much attention along with the two-dimensional diamond or diamane [1], which is of a great interest currently. Numerous theoretical studies have outlined the prospects for the application of this nanostructure in nanooptics and nanoelectronics as ultra-hard coatings with broad-range optical transparency, host material for single-photon emitter, defect center for quantum computing, etc. [2]. However, the synthesis of a 2D diamond is the most challenging field since unlike graphene and many other two-dimensional materials, diamane cannot be cleaved from the bulk. Moreover, a thermodynamic analysis shows that a few-layered diamond film without a coverage layer is simply unstable and decomposes into multilayered graphene [3,4] because the diamond surface energy is higher than the one of graphite. This conclusion is well supported by experiment [5] where the direct pressure in diamond anvil cells was used to induce conversion of the whole graphene-flake while the diamondization pressure was much higher than in the bulk case, and instability of the formed diamondized film was apparent after pressure release.

The most promising way to obtain a two-dimensional diamond seems to be the use of graphene as a precursor, by deposition of reference atoms (e.g., hydrogen) on its surface. In this case the thermodynamic stability of the material is reversed, the previously unstable diamond film becomes energy favorable, and graphene layers tend to bond to each other [4]. Despite a number of encouraging experimental results [6,7,8,9,10] confirming such predictions, the question of diamane synthesis is far from being resolved. Indeed, the nucleation of the diamane in graphene is hindered by the high stability of the graphene π-system resisting attachment of reference atoms. As a result, only two layers of graphene can be connected relatively easily, and only in the case of using hydrogen plasma as a hydrogen source [4,11,12,13]. In the case of using H_2_, we can expect the appearance of a significant nucleation barrier which can be overcome only by high pressure and temperature, for instance, in the case of single-layer graphene hydrogenation is problematic [13,14].

However, the real structure of graphene contains structural defects that can be used as nucleation centers which may allow the synthesis of diamane under less severe conditions. Here we investigate such an effect in detail. For this purpose, we study one of the most common structural defects in graphene and reveal their impact on diamond nucleation.

## 2. Computational Details

An isolated bigraphene cluster was chosen as the model system. The edge atoms of bigraphene were passivated by hydrogen atoms. The flake diameter was about 30 Å which made it possible to neglect the influence of edge effects on the process of nucleation in the flake center. For geometry relaxation and calculation of the structure energy, the DFTB method was used [15]. In this work we employed parameters for C and H atoms from the Third-Order Parametrization for Organic and Biological Systems (3OB) set [16]. Calculations were performed via DFTB+ package [17]. All atomic positions were relaxed using the conjugate gradient method. The convergence cutoff for the self-consistent evaluation of charges was 10^−5^ a.u. while maximum force components were found less than 10^−4^ Hartree/Bohr.

The bonding energy of the H_2_ molecule calculated in the framework of DFTB differs from the reference DFT data. Thus, we used in our calculations the reference value of difference (0.23 eV/atom) between the H-H and C-H binding energy for H_2_ molecule and infinite graphane [14], respectively.

## 3. Results and Discussion

The growth of the diamond phase in multilayer graphene has a nucleating character [11]. This means that the final structure is determined by the initial stages of diamond core formation and can be affected by the imperfections involved in the nucleation. To study this problem it is necessary to consider the step-by-step growing of the diamond in graphene by subsequent attachment of H atoms. We considered small groups of H on graphene starting from 1, 2, and 3 atoms and gradually increasing up to large clusters. For the cluster of *n* chemisorbed H atoms the average formation energy ε*_b_* (*n*) is $\varepsilon_{b}\left(n\right)=\frac{1}{n}\left(E_{g}+n\varepsilon_{H}-E_{nH@g}\right)$, where *E_g_* is the energy of either monolayer or bilayer graphene substrate, ε*_H_* is the energy of a single H atom, and *E_nH_*_@*g*_ is the total energy of the hydrogenated structure.

**Figure 1:**
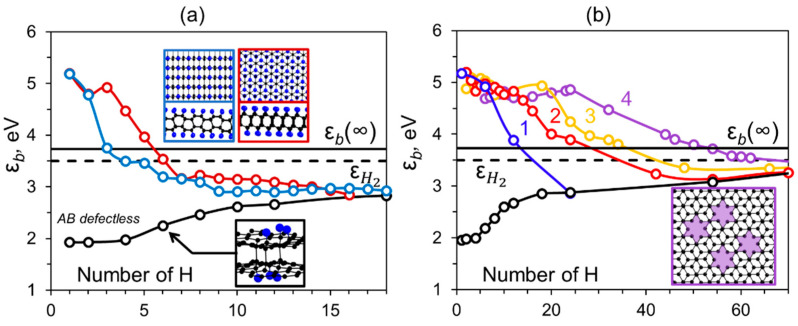
(**a**) Average binding energy ε*_b_* (*n*) as a function of numbers of atoms in H cluster for bilayer graphene contained a vacancy in one layer with AB (red line) and AA’ (blue line) stacking. In the inset, the top and side views of atomic structures of infinite cubic and hexagonal diamane are presented framed by the corresponding color. Also the initial stage of diamane nucleation in perfect AB stacked graphene is presented (framed by black). (**b**) Average binding energy as a function of numbers of atoms in H cluster for AB bilayer graphene contained 1 (blue line), 2 (red line), 3 (yellow line), and 4 (purple line) cross layer vacancies. In the inset top view of graphene with 4 vacancies agglomerate is presented framed by the corresponding color. H bonding energies in the H_2_ molecule ($\varepsilon_{H_{2}}$) and in infinite diamane (ε*_b_* (∞)) are marked by dashed and solid horizontal lines, respectively. The ε*_b_* (*n*) dependence for hydrogen on the surface of defectless AB bilayer graphene is shown by black.

Simulation of the diamond phase growth in the graphene monolayer and bilayer revealed a fundamental difference despite the similar trend of the formation energy on the number of attached hydrogen atoms [11]. The attachment of H atoms to bilayer graphene leads to the formation of interlayer C-C bonds due to the pyramidalization of adjacent hydrogenated C atoms. However, the absolute values of hydrogen binding energy at the initial stages of nucleation are sufficiently (by ~ 1 eV) lower than the same value for a monolayer (see Figure S1). This indicates much less stability of the formed diamond core which should not be a significant issue in the case of hydrogen plasma treatment of graphene because the nucleation proceeds barrier-free in any case. However, if the hydrogen source is taken in its molecular form (which is more accessible for the experiment) the situation changes dramatically. If we compare the energy of the formed C-H bonds on the bigraphene with the bonding energy in H_2_ molecule (Figure 1, horizontal dashed line) it becomes obvious that hydrogen adsorption from the molecular form is energetically unfavorable up to the large size of the diamond core, particularly more than 70 atoms in the case of perfect bilayer graphene. Indeed, the binding energy for H atoms is weaker than the H_2_ bond for all considered hydrogenation steps with a very slow tendency to a fully hydrogenated case (Figure 1, horizontal solid line). This unfavorably distinguishes the hydrogenation process of bigraphene from the case of the graphene monolayer where the diamond core becomes stable after 16 hydrogen atoms [14].

Despite the lower energy of C-H bonds in bilayer graphene, the formation of the diamond phase occurs almost immediately after the adsorption of 6 hydrogen atoms, i.e., 3 in each layer (Figure S2). This is critically important to change the hybridization of the carbon atoms in the first coordination sphere (see the inset in Figure 1a). The geometry of the first coordination sphere determines the way of a diamane formation. Therefore, it is important to accurately determine, or adjust, the structure of the nucleus at the initial stages of diamane formation. Moreover, even for the same bigraphene stacking it is possible to form diamond films with various surfaces [18]. If we consider that the stacking energy profile in the bilayer graphene is smooth [19], it can provide us control over the final structure of the diamond film.

The diamondization of defectless bigraphene is hindered by the stable π-system of sp^2^-hybridized carbon. However, the presence of structural imperfections potentially can promote both the functionalization of carbon and the connection between graphene layers. Here we studied commonly considered graphene point defects: vacancy, Stone–Wales, as well as linear defect, grain boundary.

The monovacancy defect is attractive for hydrogenation which yields the initially strong C-H bonds. The high activity of carbon atoms near the vacancy allows rapid formation of a diamond core when the bonding of layers occurs already after the adsorption of 3–4 hydrogen atoms. The first three hydrogen atoms in the case of AB stacking passivate the dangling bonds of the vacancy atoms (Figure S3). In the case of AA’ packing, only two of the three vacancy atoms are passivated, after which the third hydrogen atom attaches to the defect-free neighbored graphene layer, resulting in the formation of the first C-C interlayer bond. Next, we studied different patterns of diamond core formation in the case of AB and AA’ bigraphene stacking, as seen in Figure 1a. While in the case of AB packing all neighboring vacancy atoms are passivated with hydrogen atoms, in the case of AA’ one of the atoms binds to the atom of nearby perfect bigraphene layer. This effect can be explained by the curvature of the defective graphene sheet which changes the interlayer distance C–C. This leads to more rapid formation of the diamond core which further results into the formation of $\left(10\bar{1}0\right)$ lonsdaleite surface. If we take into account that AA’ and AB bigraphene stackings have very close energy and can occur within the same film, we can conclude that there is a probability of lonsdaleite formation via chemically induced phase transition instead of previously reported cubic diamond for the case of perfect bigraphene [11].

However, it should be noted that the energy of C-H bonds formed near the single vacancy quickly tends to a corresponding dependence obtained for perfect graphene already after deposition of 15 hydrogen atoms indicating rapidly decaying influence of the active center on the graphene structure. The attachment of such a number of H atoms forms a diamond core of sufficient size to spread beyond the defective region of the bigraphene repeating the corresponding hydrogen arrangement scheme for the perfect case and, consequently, preserving the geometry of the corresponding diamond film (cubic and hexagonal diamond for AB and AA’ stacked bigraphene, respectively).

The presence of several nearby vacancies can facilitate diamond formation. In contrast to the vacancy located only in one layer, we considered the agglomeration of cross layer vacancies in bilayer AB stacked graphene which can be obtained by its irradiation with low-energy ion beams of high density. In this structure, the defects affect each other. It leads to the formation of a reactive area between them which easily binds hydrogen atoms and forms interlayer bonds. We considered an agglomerate of vacancies separated from each other by about 5 Å, as seen in Figure 1b. After full passivation of the atoms in the first coordination sphere of the vacancy (binding energy 4.6–4.9 eV), passivation occurs in the region between the vacancies producing a fully hydrogenated area (Figure S4). After this step, hydrogen adsorbs on the outer perimeter of the agglomerate forming a hydrogenation front that spreads uniformly in all directions. As can be seen from Figure 1b, already after the hydrogenation of the second coordination sphere, the C-H bond energy is equivalent to the corresponding value obtained for perfect graphene. This also confirms the local influence of the defects on the phase transition processes in the bilayer graphene. As the number of nearby vacancies increases, the reactive region enlarges. It results in the shift of ε_*b*_ (*n*) intersection with $\varepsilon_{H_{2}}$ from 15 atoms for 1 vacancy (blue line in Figure 1b) to 65 hydrogen atoms for the agglomerate of 4 vacancies (purple line in Figure 1b), respectively. For the latter case, the average C-H binding energy differs only slightly from $\varepsilon_{H_{2}}$.

**Figure 2:**
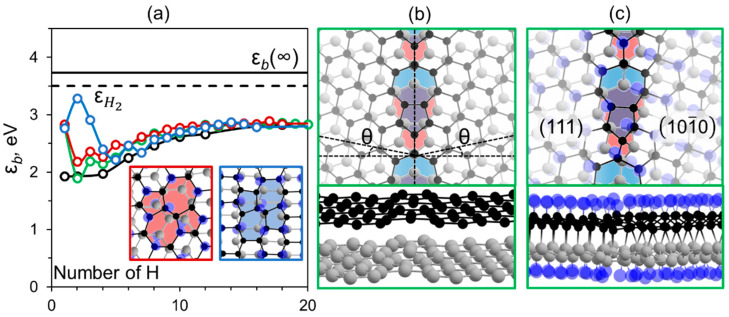
(**a**) Average binding energy per H for all clusters considered as a function of their size, *n,* for bilayer graphene containing SW defect in one layer with AB (red line), AA’ (blue line) stacking, as well as for polycrystalline bilayer graphene containing grain boundary (green line). In the inset the hydrogenated SW defect in cubic and hexagonal diamane are presented framed by the corresponding color. H bonding energies in the H_2_ molecule ($\varepsilon_{H_{2}}$) and in the infinite diamane (ε*_b_* (∞)) are marked by dashed and solid horizontal lines, respectively; (**b**) the atomic structure of polycrystalline bilayer graphene containing symmetrically inclined grains by θ = 11.5° with 5|7 defects highlighted by blue and red colors corresponding to first and second layer, respectively; (**c**) atomic structure of polycrystalline diamane produced by hydrogenation of graphene presented in (**b**) consisting of grains with cubic diamond and lonsdaleite structures.

In the case of the Stone–Wales (SW) defect commonly observed in graphene [20], we found that the energy of the initially formed C-H bonds is significantly lower than that in the case of the vacancy defect. Nevertheless, it is higher compared with the perfect surface case, see Figure 2a. The atoms of this defect are displaced from the plane which favors hydrogen adsorption and bond formation between the graphene layers. The adsorption of just two hydrogens on both bigraphene surfaces leads to the formation of the interlayer bond (Figure S5). The SW defect facilitates diamondization both in the case of AB and AA’ stacked graphene with the formation of cubic and hexagonal diamond, respectively. In the latter case, the bonding of the first hydrogen atoms leads to the binding energy increase due to the favorable adsorption of hydrogen onto carbon atoms shared by the 5- and 7-member rings [21]. After the adsorption of hydrogen atoms onto the second coordination sphere, the impact of the defect on the energy of adsorption almost vanishes and the character of binding energy becomes almost the same as in the perfect case.

Note that the Stone–Wales defect is a constituent part of the grain boundary (GB) in polycrystalline graphene connecting the graphene domains with different orientations [22]. Since stacking of bigraphene defines surface orientation and even symmetry of the produced diamond film [2], layer connection of polycrystalline bigraphene can lead to polycrystalline two-dimensional diamond consisting of grains with different surfaces. Finally, since C-H bonding at the interface is more favorable than in the case of ideal bigraphene we can assume that hydrogen deposition occurs first at the GB atoms and only then propagate in both directions connecting diamond films of different orientations in the same structure. We considered such a case in the example of the polycrystalline bilayer graphene with grains misoriented by the angle of 11.5° (Figure 2b). It was found that the C-H bonds formation in such a structure is the same as in the case of the Stone–Wales defect, as seen in Figure 2a (green line). As we expected, the initial hydrogenation occurs through the grain boundary (Figure S6) and further diamondization front spreads parallel in both directions. We noted that the energy trend of the latter region is close to that of perfect diamane. Such a process finally leads to a fully diamondized film composed of grains. This film consists of the connection of cubic and hexagonal 2D diamonds with surfaces (111) and $\left(10\bar{1}0\right)$, respectively, as seen in Figure 2c.

Thus, hydrogenation appears to be a prospective way to obtain a specific two-dimensional diamond structure that combines different surfaces. The grain boundary energy of the studied junction is ~1.3 eV/Å, which is only slightly higher than other considered two-dimensional carbon interfaces, namely graphene (<0.4 eV/Å [22]) and graphene/graphane (1.01 eV/Å [14]).

## 4. Conclusions

In summary, we found that type and concentration of structural defects can sufficiently impact the initial stages of diamond nucleation. At the same time, it does not influence further diamond formation. Defects impact on C-H bonding strength is disappearing at the second coordination sphere already. We show that vacancies agglomeration (that can be produced by low energy ion irradiation) can sufficiently expand the reactive region which vanishes the nucleation barrier for the first stages of nucleation. Stone–Wales defects impact is lower but promotes the hydrogenation and bonding of the graphene layers. We show that 1D defect (dislocation) not only facilitates the diamondization but also may lead to the appearance of 2D diamond consisting of chemically connected grains of different crystallographic orientations. Therefore, polycrystalline graphene usually observed in the experiment can produce specific 2D diamond polycrystals containing different surfaces. Even hexagonal and cubic 2D diamonds can coexist together in the same film with grain boundary energy comparable with the same values for other two-dimensional carbon structures.

Our study can be further expanded by more detailed investigation of the thermodynamic stability of formed diamond clusters and the explicit calculations of nucleation barrier dependence on a pressure. Other possible structural defects (divacancies, 5|8|5, 555|777, etc.) as well as multilayer graphene (more than two layers) should be also considered in further work.

We believe that the present study will help in further research on the diamondization of multilayer graphene and producing of new carbon nanomaterials with tunable properties for various applications.

## Data Availability

Not applicable.

## Supplementary Materials

The following supporting information can be downloaded at: https://www.mdpi.com/article/10.3390/nano12223983/s1, Figure S1: Average binding energy ε*_b_* (*n*) as a function of the H number on the surface of perfect monolayer and bilayer graphene; Figure S2: Structures of H atoms adsorbed on perfect bilayer graphene with AB stacking; Figure S3: Structures of H atoms adsorbed on bilayer graphene contained vacancy defect in one layer with AB and AA’ stacking; Figure S4: Structures of H atoms adsorbed on bilayer graphene contained 4 cross layer vacancies; Figure S5: Structures of H atoms adsorbed on bilayer graphene contained SW defect in one layer with AB and AA’ stacking; Figure S6: Structures of H atoms adsorbed on polycrystalline bilayer graphene contained symmetrically inclined grains by θ = 11.5°

## References

[1] Chernozatonskii L.A., Sorokin P.B., Kvashnin A.G., Kvashnin D.G. (2009). Diamond-like C2H Nanolayer, Diamane: Simulation of the Structure and Properties. JETP Lett..

[2] Sorokin P.B., Yakobson B.I. (2021). Two-Dimensional Diamond—Diamane: Current State and Further Prospects. Nano Lett..

[3] Li H., Li J., Wang Z., Zou G. (2012). Layer Number-Dependent Structural Evolution of Two-Dimensional Diamond Films. Chem. Phys. Lett..

[4] Kvashnin A.G., Chernozatonskii L.A., Yakobson B.I., Sorokin P.B. (2014). Phase Diagram of Quasi-Two-Dimensional Carbon, from Graphene to Diamond. Nano Lett..

[5] Ke F., Zhang L., Chen Y., Yin K., Wang C., Tzeng Y.-K., Lin Y., Dong H., Liu Z., Tse J.S. (2020). Synthesis of Atomically Thin Hexagonal Diamond with Compression. Nano Lett..

[6] Tao Z., Du J., Qi Z., Ni K., Jiang S., Zhu Y. (2020). Raman Spectroscopy Study of Sp2 to Sp3 Transition in Bilayer Graphene under High Pressures. Appl. Phys. Lett..

[7] Bakharev P.V., Huang M., Saxena M., Lee S.W., Joo S.H., Park S.O., Dong J., Camacho-Mojica D.C., Jin S., Kwon Y. (2019). Chemically Induced Transformation of Chemical Vapour Deposition Grown Bilayer Graphene into Fluorinated Single-Layer Diamond. Nat. Nanotechnol..

[8] Piazza F., Cruz K., Monthioux M., Puech P., Gerber I. (2020). Raman Evidence for the Successful Synthesis of Diamane. Carbon.

[9] Pimenta Martins L.G., Silva D.L., Smith J.S., Lu A.-Y., Su C., Hempel M., Occhialini C., Ji X., Pablo R., Alencar R.S. (2021). Hard, Transparent, Sp3-Containing 2D Phase Formed from Few-Layer Graphene under Compression. Carbon.

[10] Colin M., Chen X., Dubois M., Rawal A., Jun Kim D. (2022). F-Diamane-Like Nanosheets from Expanded Fluorinated Graphite. Appl. Surf. Sci..

[11] Erohin S.V., Ruan Q., Sorokin P.B., Yakobson B.I. (2020). Nano-Thermodynamics of Chemically Induced Graphene-Diamond Transformation. Small.

[12] Zhu L., Hu H., Chen Q., Wang S., Wang J., Ding F. (2011). Formation and Electronic Properties of Hydrogenated Few Layer Graphene. Nanotechnology.

[13] Antipina L.Y., Sorokin P.B. (2015). Converting Chemically Functionalized Few-Layer Graphene to Diamond Films: A Computational Study. J. Phys. Chem. C.

[14] Lin Y., Ding F., Yakobson B.I. (2008). Hydrogen Storage by Spillover on Graphene as a Phase Nucleation Process. Phys. Rev. B.

[15] Elstner M., Porezag D., Jungnickel G., Elsner J., Haugk M., Frauenheim T., Suhai S., Seifert G. (1998). Self-Consistent-Charge Density-Functional Tight-Binding Method for Simulations of Complex Materials Properties. Phys. Rev. B.

[16] Gaus M., Goez A., Elstner M. (2013). Parametrization and Benchmark of DFTB3 for Organic Molecules. J. Chem. Theory Comput..

[17] Hourahine B., Aradi B., Blum V., Bonafé F., Buccheri A., Camacho C., Cevallos C., Deshaye M.Y., Dumitrică T., Dominguez A. (2020). DFTB+, a Software Package for Efficient Approximate Density Functional Theory Based Atomistic Simulations. J. Chem. Phys..

[18] Kvashnin A.G., Sorokin P.B. (2014). Lonsdaleite Films with Nanometer Thickness. J. Phys. Chem. Lett..

[19] Reguzzoni M., Fasolino A., Molinari E., Righi M.C. (2012). Potential Energy Surface for Graphene on Graphene: Ab Initio Derivation, Analytical Description, and Microscopic Interpretation. Phys. Rev. B.

[20] Banhart F., Kotakoski J., Krasheninnikov A.V. (2011). Structural Defects in Graphene. ACS Nano.

[21] Duplock E.J., Scheffler M., Lindan P.J.D. (2004). Hallmark of Perfect Graphene. Phys. Rev. Lett..

[22] Liu Y., Yakobson B.I. (2010). Cones, Pringles, and Grain Boundary Landscapes in Graphene Topology. Nano Lett..

