# Supplementary material for: The Role of Structural Defects in the Growth of Two-Dimensional Diamond from Graphene
Source: Nanomaterials (Basel). 2022 Nov 12;12(22):3983. doi: 10.3390/nano12223983 (PMC9698712; doi:10.3390/nano12223983)
Supplement: Supplementary file 1 [file nanomaterials-12-03983-s001.zip › nanomaterials-2035999-supplementary.pdf]

Supplementary Materials

The Role of Structural Defects in the Growth of Two-Dimensional Diamond from Graphene

Liubov A. Varlamova, Sergey V. Erohin and Pavel B. Sorokin *

[1] Laboratory of Digital Material Science, National University of Science and Technology MISIS, 4 Leninskiy prospekt, 119049 Moscow, Russia

* Correspondence: pbsorokin@misis.ru

S1. The dependence of the diamond formation energy in the graphene monolayer and bilayer on the number of attached hydrogen atoms

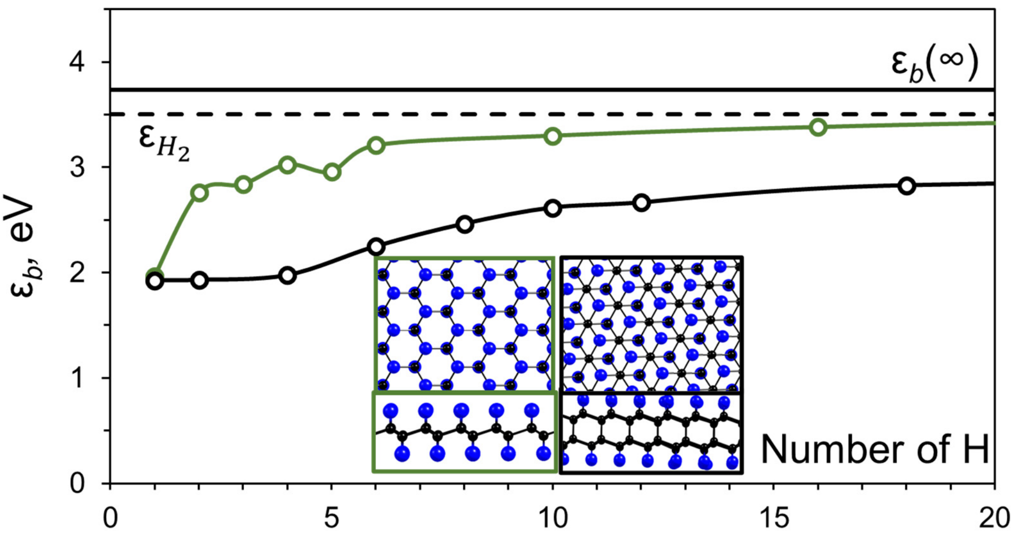

Figure S1. Average binding energy $\varepsilon_b(n)$ as a function of the H number on the surface of perfect monolayer (green line) and bilayer graphene (black line). In the insets the top and side views of atomic structure of infinite graphane and diamane are presented framed by the corresponding colors. H bonding energies in the H_2 molecule (ε_{H_2}) and in infinite graphane (diamane) ($\varepsilon_b(\infty)$) are marked by dashed and solid horizontal lines, respectively

S2. Structures of H atoms adsorbed on bilayer graphene

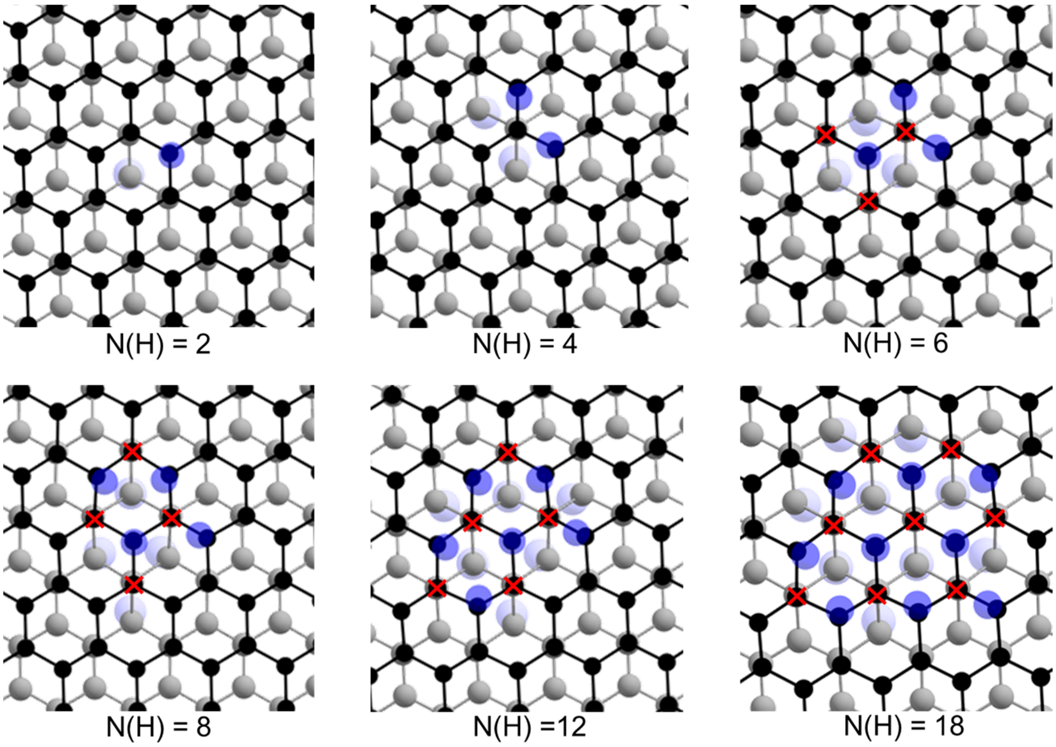

Figure S2. Structures of H atoms adsorbed on perfect bilayer graphene with AB stacking. C-C bond between neighbored layers is depicted by red crosses

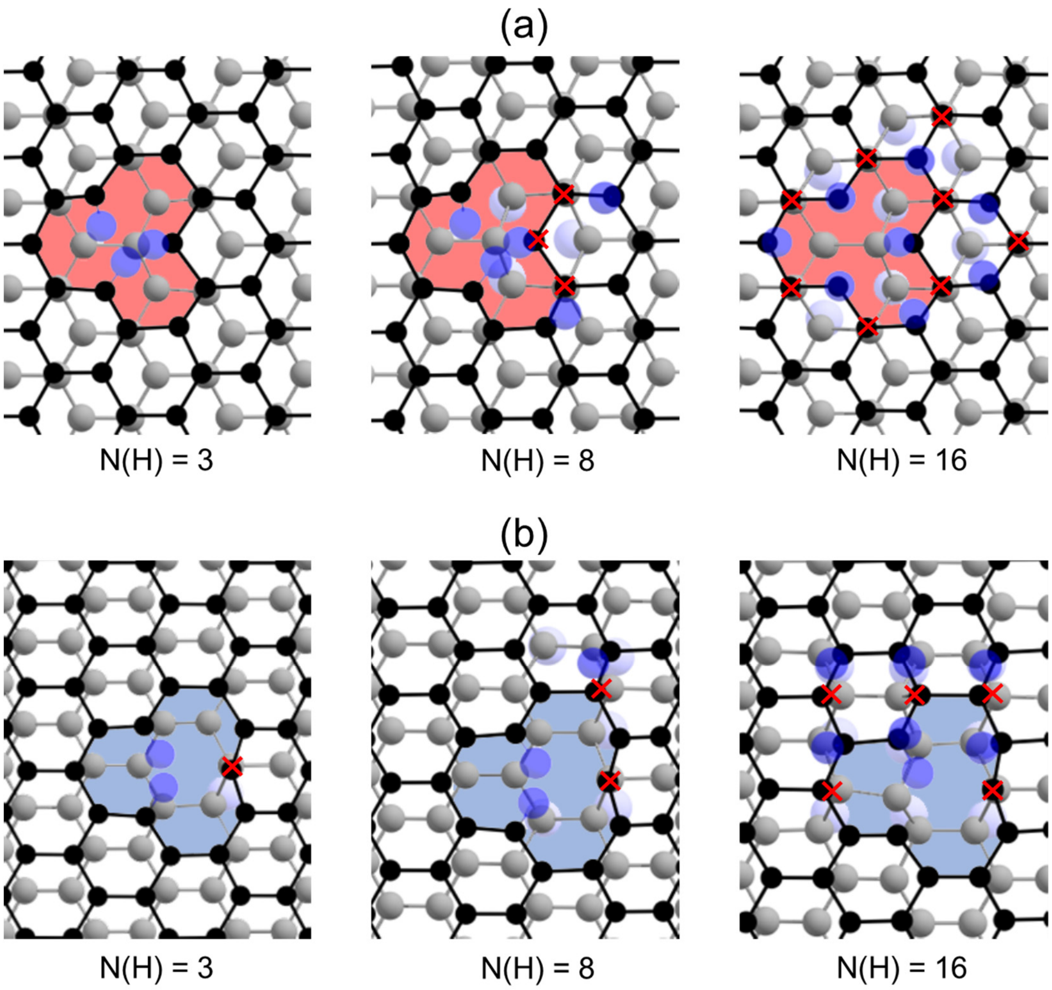

Figure S3. Structures of H atoms adsorbed on bilayer graphene contained vacancy defect in one layer with a) AB and b) AA′ stacking with defect highlighted by red and blue color, respectively. H atoms adsorbed on the opposite sides are marked by dark and light blue colors, C-C bond between neighbored layers is depicted by red crosses

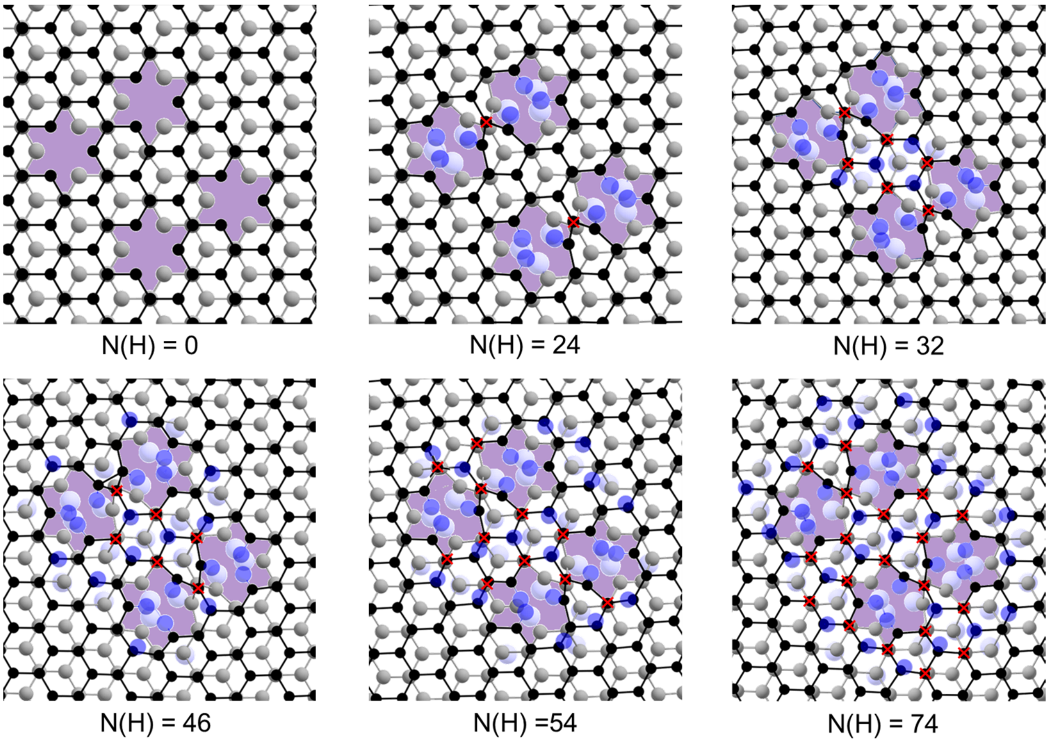

Figure S4. Structures of H atoms adsorbed on bilayer graphene contained 4 cross layer vacancies (highlighted by purple). H atoms adsorbed on the opposite sides are marked by dark and light blue colors, C-C bond between neighbored layers is depicted by red crosses

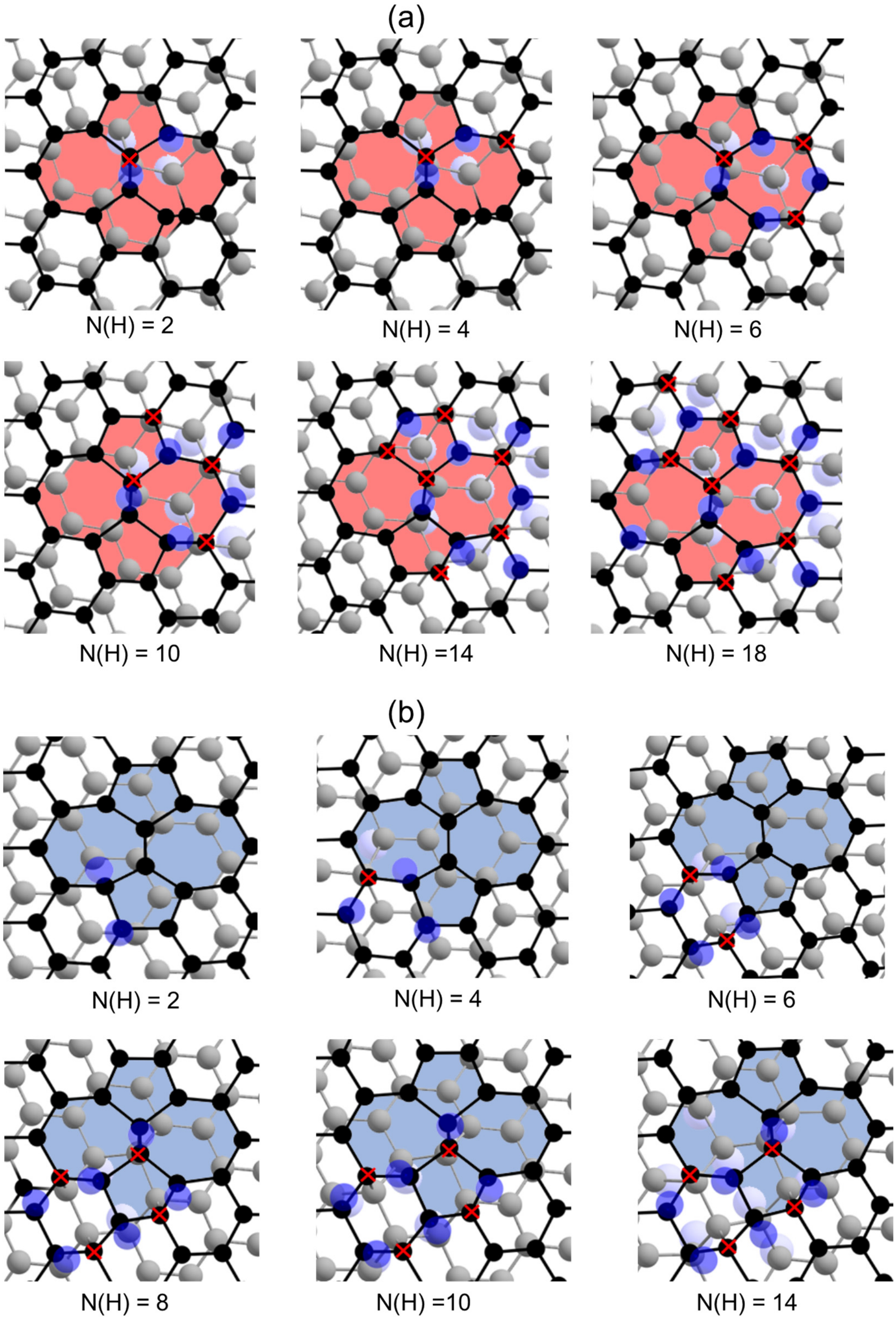

Figure S5. Structures of H atoms adsorbed on bilayer graphene contained SW defect in one layer with a) AB and b) AA′ stacking with defects highlighted by red and blue color, respectively. H atoms adsorbed on the opposite sides are marked by dark and light blue colors, C-C bond between neighbored layers is depicted by red crosses

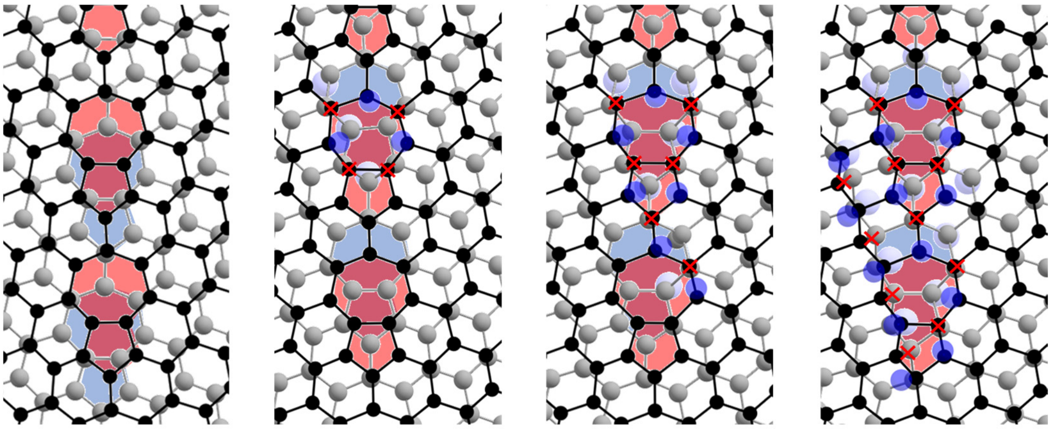

Figure S6. Structures of H atoms adsorbed on bilayer polycrystalline graphene contained symmetrically inclined grains by θ = 11.5 º highlighted by blue and red colors corresponding to first and second layer, respectively. H atoms adsorbed on the opposite sides are marked by dark and light blue colors, C-C bond between neighbored layers is depicted by red crosses
